# Patterns in Temporal Variability of Temperature, Oxygen and pH along an Environmental Gradient in a Coral Reef

**DOI:** 10.1371/journal.pone.0085213

**Published:** 2014-01-08

**Authors:** Òscar Guadayol, Nyssa J. Silbiger, Megan J. Donahue, Florence I. M. Thomas

**Affiliations:** 1 Hawai‘i Institute of Marine Biology, School of Ocean and Earth Science and Technology, University of Hawai‘i at Mānoa, Kāne‘ohe, Hawai‘i, United States of America; 2 Department of Biology, University of Hawai‘i at Mānoa, Honolulu, Hawai‘i, United States of America; University of Waikato (National Institute of Water and Atmospheric Research), New Zealand

## Abstract

Spatial and temporal environmental variability are important drivers of ecological processes at all scales. As new tools allow the *in situ* exploration of individual responses to fluctuations, ecologically meaningful ways of characterizing environmental variability at organism scales are needed. We investigated the fine-scale spatial heterogeneity of high-frequency temporal variability in temperature, dissolved oxygen concentration, and pH experienced by benthic organisms in a shallow coastal coral reef. We used a spatio-temporal sampling design, consisting of 21 short-term time-series located along a reef flat-to-reef slope transect, coupled to a long-term station monitoring water column changes. Spectral analyses revealed sharp gradients in variance decomposed by frequency, as well as differences between physically-driven and biologically-reactive parameters. These results highlight the importance of environmental variance at organismal scales and present a new sampling scheme for exploring this variability *in situ*.

## Introduction

The spatial and temporal distribution of aquatic organisms is tightly coupled to underlying physical, chemical, and biological changes that are distributed unequally over a wide range of scales (e.g. [1], [2]). Most of this variability is concentrated in large-scale phenomena, such as seasonal cycles or latitudinal gradients. Thus, the effects of low frequency environmental variability, expressed in terms of means, trends and seasonality, have received more attention than variance at high frequencies (e.g. [3]). However, behavioral and physiological responses of individuals occurs mainly at small scales (e.g. [4]–[9]), and environmental variability at high frequencies may be important to structuring aquatic systems. Therefore, characterizing the whole spectrum of variance experienced by organisms is important if we want to understand how responses of individuals to perturbations scale up into an ecosystem response [10]. Focusing on smaller scale variability is particularly relevant in the present context of environmental change because the scale and frequency of variation is expected to shift along with mean changes in global climate [11].

The development of faster, more accurate and affordable autonomous sensors has triggered studies describing patterns of natural variability in the framework of global change, particularly for pH [12]–[15]. A number of these studies have focused on determining ranges of biologically relevant variables across contrasting marine environments [13] or in seasonal and/or diurnal variability [14], [16]–[17], but intermediate and smaller scales have received little attention. Thus, studies exploring the whole spectrum of temporal environmental variability, and how this changes along spatial gradients, are lacking. Such multiscale studies can help us understand the distribution of organisms and can provide a background for testing, *in situ,* hypotheses about how organisms will acclimate and adapt [18] to upcoming changes in environmental variability.

Tropical coral reefs are particularly appropriate for an investigation of high-frequency environmental fluctuations because dramatic changes in temporal variability can occur over very short distances for several reasons. First, coastal systems are boundaries between ocean, land, and atmosphere and are subjected to a wide array of fluctuations from all three sources. Second, reef shorelines can be very abrupt, and steep physical and chemical gradients are established across changes in depth. Concurrent with these physical gradients are gradients in primary productivity, which can drive fluctuations in oxygen and pH over relatively small temporal and spatial scales. Third, tropical systems are characterized by a relatively low seasonality and strong diurnal forcing; therefore, high-frequency variability may be comparatively more important than in temperate environments. In addition, the need to understand patterns of natural variability is particularly acute on coral reefs, because corals show narrow tolerance levels and are sensitive to global change [19]–[21]. Reef organisms may undergo acclimatization to small-scale spatial and temporal variation in the environment [22]–[23]. Thus, describing the levels of natural variability these organisms are facing at all scales is critical for predicting their resilience to global change [14]. Here we examine the spatial distribution of the whole spectra of environmental fluctuations across a coral reef in Kāne‘ohe Bay, Hawai‘i, using a novel spatial and temporal sampling scheme that combines short time series from sites distributed across a transect, with a long-term time series from an adjacent permanent station.

## Methods

### Site Description

Moku o Lo‘e (Coconut Island), home to the Hawai‘i Institute of Marine Biology, is a small island of about 12 ha located in Kāne‘ohe Bay, a tropical semi-enclosed embayment at the eastern shore of the island of O‘ahu in the Hawaiian archipelago. It is situated on the southern basin of the bay lagoon, where water residence time is relatively long [24]–[25] and advection is minimal. The biochemistry of this basin is heavily influenced by land, and subject to episodic releases of nutrients [26]–[27].

Seasonality is defined by dry (May to October) and rainy (October to April) seasons. NE trade winds are prevailing throughout the year, but occasionally intense south “Kona” winds occur in winter. A weak thermocline is usually established in summer at a depth of about 8.5 m [28].

Diurnal and semidiurnal components are the only significant tidal constituents [29]. Mean and maximum tidal ranges are 0.7 and 1.1 m, respectively, and currents are generally mild and tidally driven [25], [30].

### Spatio-temporal Sampling Design

In order to explore distribution in variance across spatial and temporal scales, we established 21 random-stratified sampling locations along a ∼32 m reef flat-to-reef slope transect across a fringing reef. At each site along the transect, two-week long times series of environmental parameters were obtained non-simultaneously throughout a period of 11 months. At an adjacent long-term station, we obtained a similar time series for the entire experimental period. This is an approximation to an idealized (albeit inefficient and expensive) setup with a grid of sensors deployed simultaneously.

**Figure 1:**
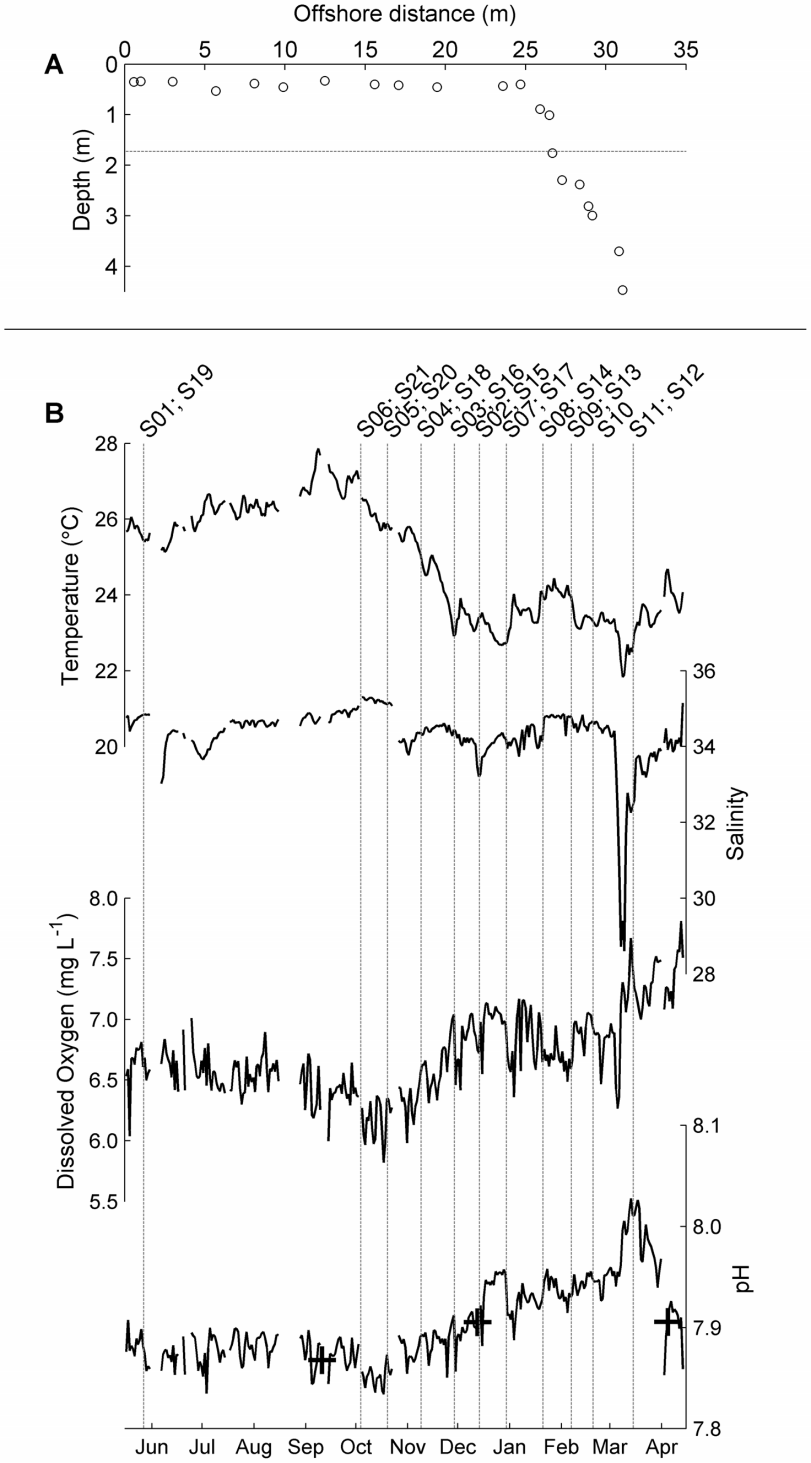
Timing and position of sensor deployments. (A) Position of sites in the reef-flat to reef-slope transect on distance to shore-depth axes. Sites are numbered consecutively (S1–S21) from left to right. Horizontal dotted line marks the depth at which the long-term time series was located. (B) Time series plots of daily averaged temperature, salinity, dissolved oxygen concentration and pH from the long-term station. Vertical dotted lines mark the starts of the two-weeks deployments along the transect. Crosses on top of the pH time series correspond to in situ water measurements of pH. The names of the sites used in the deployments are shown on top.

The transect was oriented eastward off the southeastern shore of Coconut Island (N21°25.975′, W157°47.175′). Starting 0.5 meters from shore, the transect ran along the reef flat for ∼24 m and then down the reef slope for another ∼7 m to a depth of 4.5 m (Fig. 1A). Sites were stratified between the reef flat and reef slope, with sites S1–S11 on the flat and S12–S21 on the slope. The transect falls mostly within the sublittoral zone, although the shallowest parts of the reef flat are occasionally exposed during the lowest tides. During each sampling period, measurements were taken simultaneously at two randomly selected locations on the transect, one in the reef slope and the other in the flat, using multi-parametric probes. The random order helped ensure that the spatial gradient along the transect was not systematically confounded by long-term temporal trends or seasonality. The instruments were deployed at 5 to 10 cm above the bottom for a minimum of 2 weeks between May 2011 and March 2012 (Fig. 1B). Each probe contained temperature, conductivity, dissolved oxygen concentration ([O_2_]) and pH sensors (Sonde 600XLM, YSI Incorporated). Only temperature, pH and [O_2_] data were used in this study. Salinity could not be used in the statistical analyses because its distribution was not normal, and the time series were spiky. Therefore, the spectral analyses resulted in flat, anomalous spectral densities.

The long-term station was located a few meters south of the transect, just off the easternmost point of Moku o Lo‘e, on a site with a shorter reef flat than the transect. It consisted of a multi-parametric instrument (Sonde 6600V2-4, YSI) with sensors for temperature, conductivity, pH and [O_2_]. As the aim was characterizing water column background conditions, rather than bottom conditions, the instrument was placed in midwater, attached facing down to a pole at a depth of ∼1.7 m over a ∼3 m deep bottom.

In addition, an acoustic Doppler current profiler (ADCP, 2.0 MHz Aquadopp Profiler, Nortek A.S.) was placed at about 14.0 meters in depth on average, a few meters offshore of the long-term station. It measured at 20 bins of 0.5 m. No specific permits were required for sensor deployments at our study site.

### Sensor Performance and Configuration

Sampling rates were limited by power and memory to ∼0.1 min^−1^, resulting in a Nyquist frequency of 3 h^−1^. Temperature sensors (YSI 6560) were thermistors with an accuracy of ±0.15°C and a resolution of 0.01°C according to manufacturer [31]. Oxygen sensors (YSI 6150 ROX) were optical probes based on the luminescence lifetime method and had ±0.1 mg·L^−1^ accuracy and 0.01 mg·L^−1^ resolution for the ranges encountered in this study [31]. Sensors for pH (pH/ORP YSI 6565) were bulb-shaped glass electrodes with ±0.2 accuracy and 0.01 resolution [31]. All multi-parametric probes were calibrated periodically in the laboratory using standard procedures and calibration solutions. The long-term station was recovered, cleaned, calibrated, and re-deployed 3 times during the study, and the probes used for the transect were calibrated 7 times. Pre-calibration measurements of commercial standard solutions were conducted to detect any relevant drifts in the sensors. In all cases the difference between the post and pre-calibration values was equal or better than the accuracy of the sensors, and thus it was concluded that no detectable drift was observed. pH sensors were calibrated in the laboratory against NBS standards, and then converted into total pH scale using CO2SYS program [32]. To further account for any undetected drift and to increase the accuracy of pH measures, we calibrated sensor values against water samples collected every six hours over 24 h periods in December and April. pH samples were analyzed using standard spectrophotometric techniques and calibrated against a Tris buffer following [33].

### Spectral Analyses

**Figure 2:**
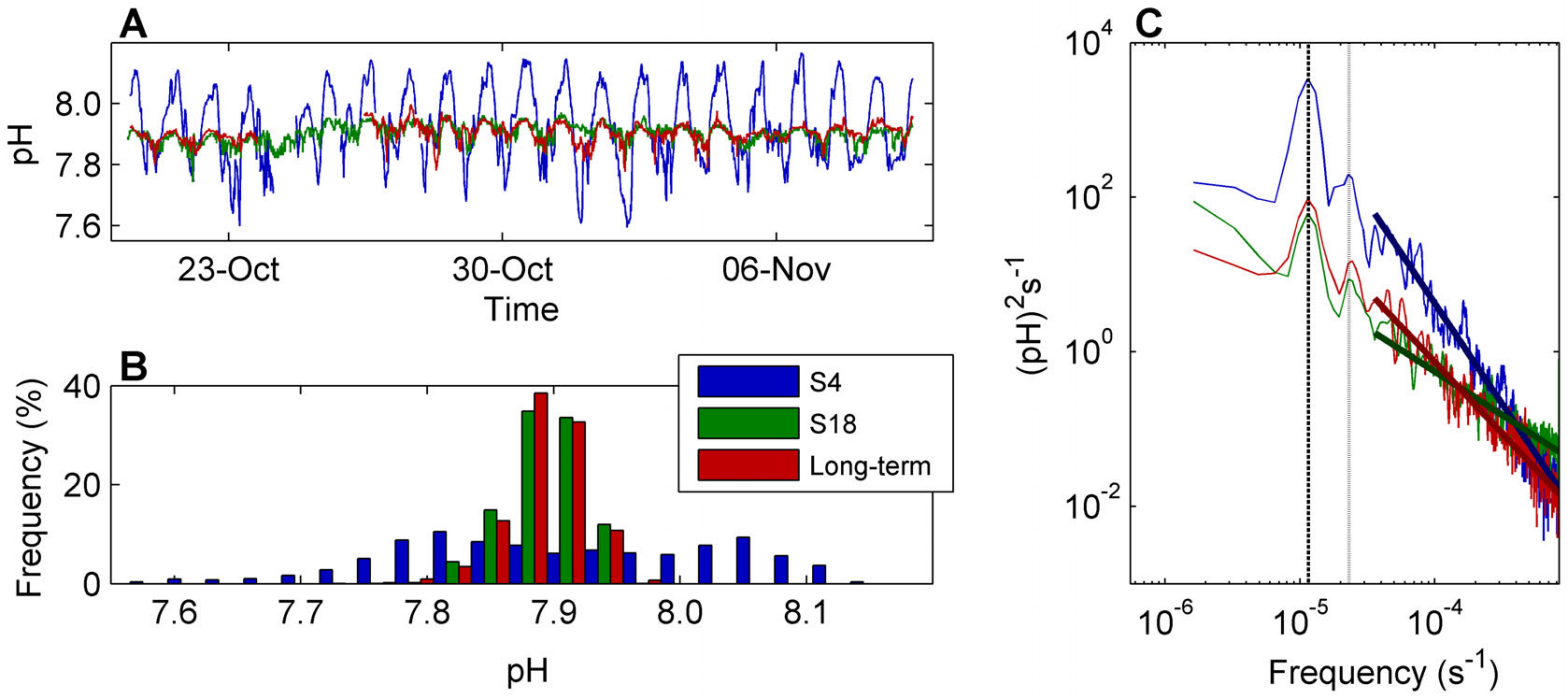
Example of 3 simultaneous time-series of pH. Simultaneous time-series of pH, taken from October 20th to November 9th 2011. The sites shown are: S4 (in blue), located 8.2 m offshore at a depth of 0.4 m, S18 (in green), located 31.6 m offshore at a depth of 3.7 m, and the long-term station (in red), located at 1.7 m depth over a bottom 3 meters deep. (A) Time series plot. (B) Histograms of frequencies of pH values during this period. (C) Power spectral densities. Darker straight lines in (C) depict the best fit power-law models between frequencies 1/8 h^−1^ and 3 h^−1^ obtained using linear least squares method. The vertical dashed and dotted lines in the spectra mark the diurnal and semi-diurnal frequencies respectively.

The distribution of variance across frequencies was explored using spectral analyses of the time series. We then compared these distributions among the sites along the spatial transect. Spectral analyses are a set of mathematical tools that transform a data series from the temporal to the frequency domain (e.g. Fig. 2A & C). Results are usually plotted as periodograms graphing the density of variance (or, depending on context, power or energy) against frequency (Fig. 2C). The integral of a density spectrum over all frequencies gives an estimate of the total variance of the signal. Similarly, cross-spectral analyses of two simultaneous time series can be used to produce periodograms of density of covariance vs. frequency, which integrate to total covariance. We examined the spectra in two ways: 1) by decomposing the variance (or covariance) over different frequency ranges, and 2) by comparing the scaling exponents of the power-law decay of variance against frequency.

The first way, decomposing the variance over different frequency ranges, provides information about the contribution of given frequencies to total variance or covariance. This approach is particularly useful to ascertain the importance of specific periodic processes, such as daily and tidal patterns, that show in a periodogram as peaks in spectral density. The decomposition of variance or covariance is accomplished by integrating the spectra over different frequency ranges. Just as the integration of the whole spectrum gives an estimation of the total variance or covariance of the signal, integrating specific ranges of frequencies within the spectra yields estimates of the contribution to total variance or covariance accounted for by each range (e.g. [34]–[35]). Based on the most prominent peaks observed in the periodograms (see Fig. 2C), the ranges used were: [0, 1/27), [1/27, 1/21), [1/21, 1/14), [1/14, 1/10), and [1/10, 3) hours^−1^. These frequency ranges are named throughout the paper as weekly (1d-2w period), daily (21–27 h), daily to half-daily (14–21 h), half-daily (10–14 h), and hourly (1/3–10 h) components respectively. Decomposition was done on raw periodograms obtained from fast Fourier transforms of the detrended time series. Raw spectra were divided by total variance or covariance to obtain the percentages reported here.

Power-law exponents of spectral density functions were used to identify patterns of variability at the highest end of frequencies resolved by our sampling, namely periods from 20 minutes to 8 hours. In this range, no significant peaks and no changes in the slope were observed. Spectral slopes are commonly used in the oceanographic literature to test for underlying physical models. Under isotropic turbulent conditions for example, and over certain frequency ranges, passive tracers such as temperature are expected to show the same −5/3 slope as kinetic energy [36]. Spectral densities were estimated using Welch’s method on the detrended signals [37], with 4-day segments and 50% overlap. To remove the effect of irradiation and diurnal and semidiurnal tidal constituents, which are overwhelmingly dominant in this system [29], a high-pass Butterworth filter with 1/10 h^−1^ cutoff frequency was applied to each series before performing the spectral analyses. Then, linear least-squares models were fitted to the log-log transformed spectral densities in the range between 1/8 h^−1^ and 3 h^−1^ after resampling the log-log to keep it evenly spaced in the frequency axis and avoid a disproportionate weight of the highest frequencies.

Fast Fourier transformations require uninterrupted time series, which was not the case in some of the deployments. To maximize the length of the usable time series, gaps ≤2 hours (i.e. 12 data points) were linearly interpolated. The minimum uninterrupted time series length to perform spectral analyses was set to 7 days.

### Statistical Analyses

The focus of this study is a spatial comparison of time series’ spectra across 21 locations. However, since only two locations were measured in any two-week period, sampling period could influence comparisons between locations. To assess the relative contribution of sampling period and spatial location to variation between time series’ spectra, we used variance components analyses (VCA) on the mean, total variance, decomposed variances, and spectral slopes of the measured environmental variables (pH, dissolved oxygen, and temperature). We fitted a random effects model with deployment date (10 levels) as random factor using REML estimation in the R package *nlme* (nlme version 3.1–105, [38]) in R (version 2.14.1, [39]). We excluded from this analysis the one sampling period when only a single block was measured (S10, Fig. 1B). This model generates two variance components: variance attributed to deployment date and residual variance attributed to location. In some cases, variance components approached zero. When fitted using traditional ANOVA estimation [40], these variance components were negative, indicating a true estimate of zero.

**Figure 3:**
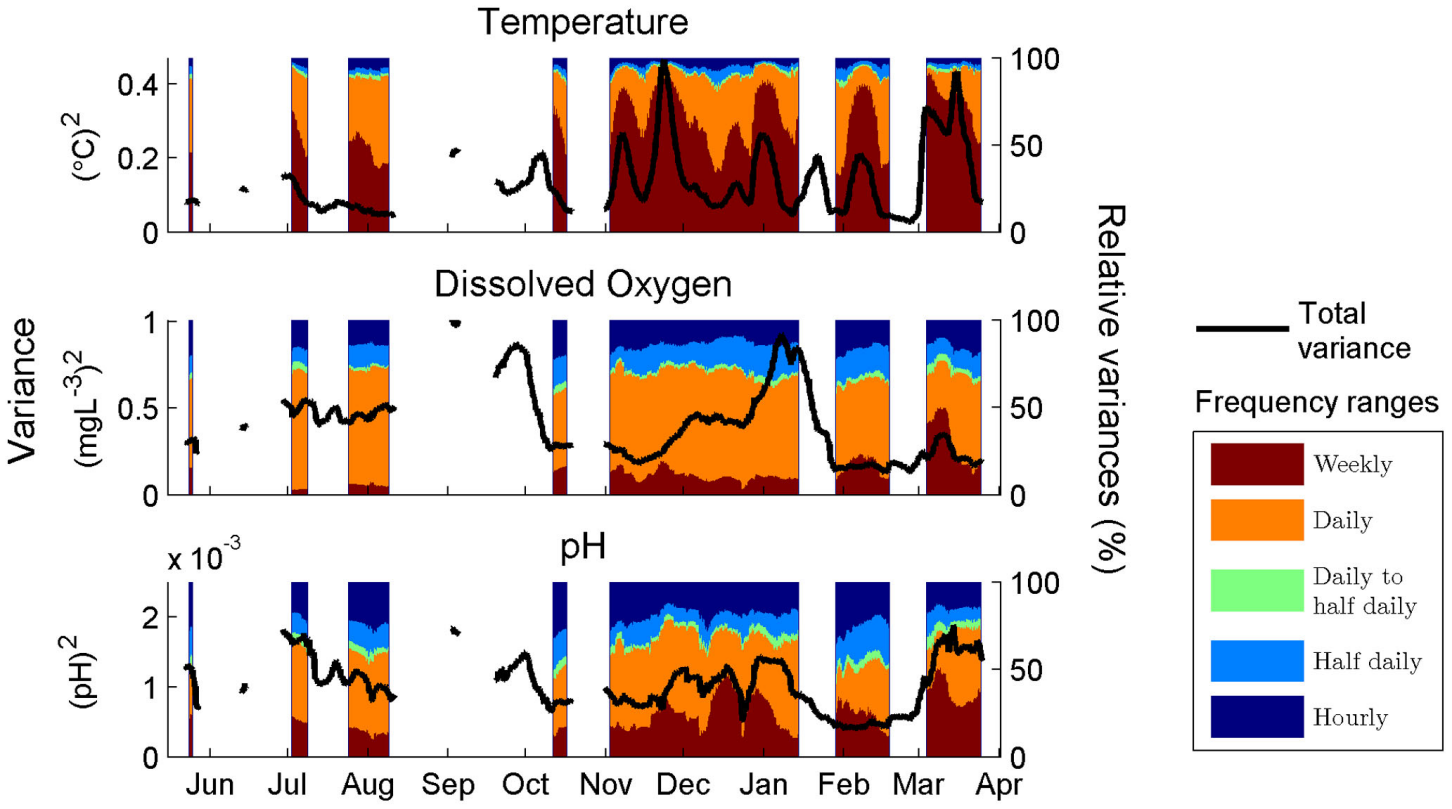
Time series of decomposed variances. Variance decomposition of temperature, dissolved O_2_ concentration and pH in the long-term time series station, obtained using a running window of two weeks. Left axes scale absolute variances (black lines), and right axes the relative contribution (in %) of each frequency range to total variance. To perform the spectral analyses the time-series need to be uninterrupted; thus short gaps in the signals resulted in the long gaps observed in the figure.

Moving averages and variances were obtained from the long-term time series using a two-week window, which makes them directly comparable to statistics computed from the two-week deployments. Segments with gaps accounting for up to 10% of the total values were accepted to compute moving statistics in figure 3.

## Results

### Variance vs. Covariance

**Figure 4:**
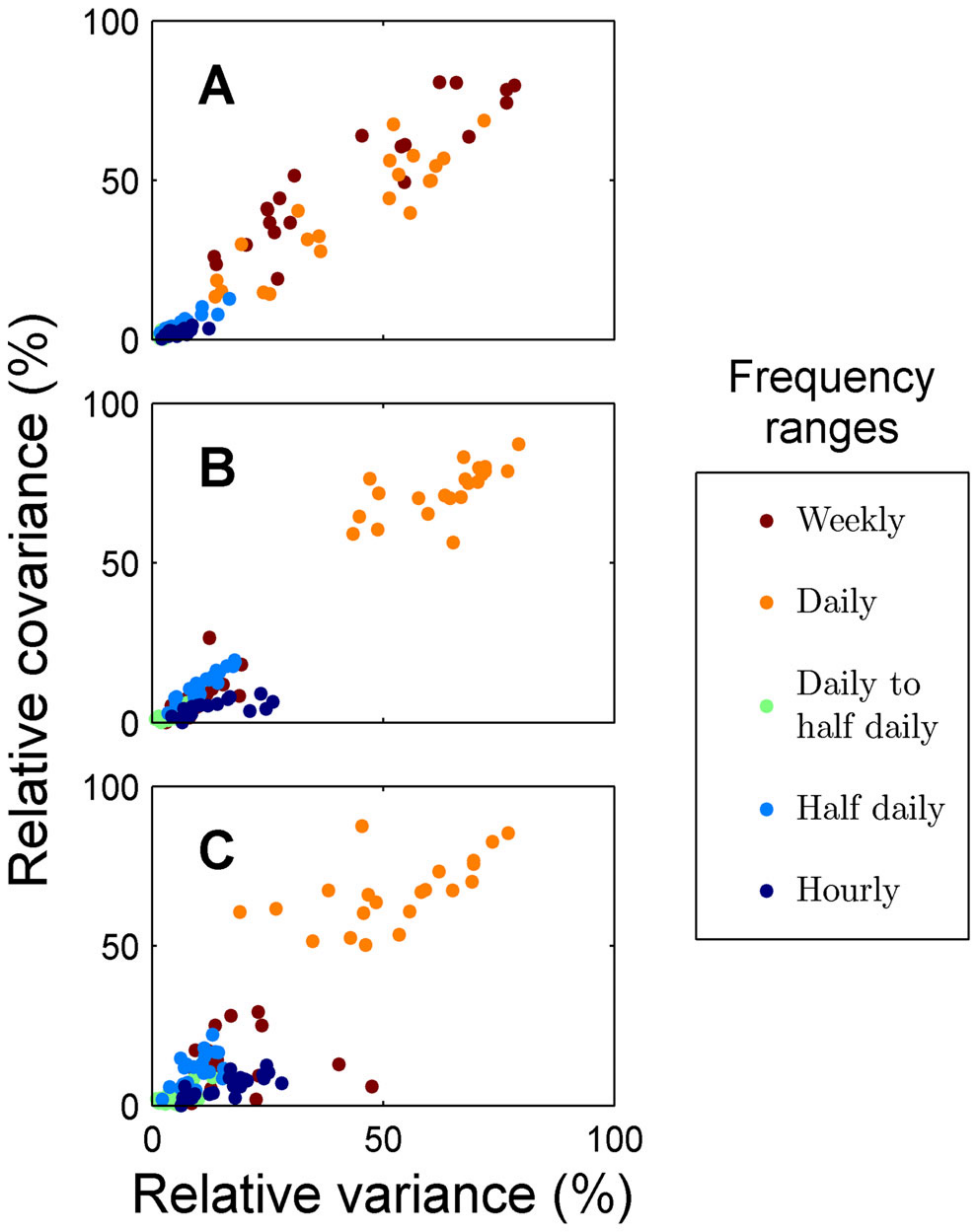
Relative decomposed covariances vs. variances. Relative decomposed covariances derived from cross-spectral analyses between transect sites and long-term station vs. relative decomposed variances derived from auto-spectral analyses of transect sites. (A) Temperature, (B) Dissolved oxygen concentration, (C) pH.

In order to discriminate spatial gradients in the statistics under study from seasonal and other long-term temporal trends, two approaches are possible. The first is using the long-term station to standardize each one of the two-week long time series along the spatial gradient. In the spectral domain, this may be accomplished using cross-spectral analyses, which partition the covariance between two series (in our case, the long-term times series vs. each one of the sites) across different frequencies. Using covariance unveils spatial patterns in high-frequency temporal fluctuations, but does not provide an easy physical interpretation. An alternative approach is using auto-spectral analyses of each short-term time series. This will yield variances, which are more easily interpreted and compared than covariances. In the present study, the patterns observed using both approaches were similar (Fig. 4), and therefore, for ease of interpretation, the results reported in the present paper correspond exclusively to auto-spectral analyses. However, the auto-spectral approach is valid only if the decomposed variances are stationary in time, or more practically, if variability in time is much lower than in space, as in the present study. Otherwise, cross-spectral analysis between each location and the long-term station must be used despite the more difficult interpretation.

Before using the auto-spectral analyses for our interpretation we tested for stationarity of the parameters derived from such analyses, using the long-term time series. As expected, none of the spectral parameters were constant throughout the period of study (Fig. 3). However, the seasonal variability in the permanent station was considerably smaller than the spatial variability along the transect. For temperature, variance at the station ranged from 0.03 to 0.46°C^2^, while variance across the transect ranged from 0.05 to 1.11°C^2^. For oxygen concentration, variance ranged from 0.13 to 1.00 mg^2^·L^−2^ at the station and from 0.19 to 6.28 mg^2^·L^−2^ along the transect. For pH, variance ranged from 4×10^−4^ to 19×10^−4^ at the station and from 7×10^−4^ to 190×10^−4^ along the transect. Overall, the range of measured variances along the transect were 2.5, 7, and 12 times greater than the range of measured variances from the permanent station for temperature, [O_2_], and pH, respectively. In relative terms, the contribution of the different frequencies to total variance was also remarkably constant throughout the year, with one exception: lowest frequency contribution on temperature variance changed considerably (Fig. 3). Spectral slopes also remained comparatively invariant though time in the long-term station, with means (±standard deviation) indistinguishable from −5/3: −1.75±0.22 for temperature, −1.64±0.15 for [O_2_], and −1.82±0.24 for pH.

The VCA comparing the relative contribution of spatial location and sampling period to the variance between time series, also showed that seasonal variability was negligible compared to spatial variability (Table 1). In general, the estimates for seasonal variability approached zero for the spectral parameters used in this study; that is, nearly all between-time-series variance was attributed to spatial location. By contrast, time explained most of the variability in mean values of temperature and [O_2_]. This is a consequence not only of the seasonal trends, but also of the lack of spatial differences in means.

**Table 1:** Variance components analyses (VCA).

		% of variability explained	
Parameter		Temporal	Spatial (Residual)
Temperature	Mean	92.1	7.9
	σ^2^_Total_	0.0	100.0
	σ^2^_Weekly_	0.1	99.9
	σ^2^_Daily_	0.0	100.0
	σ^2^_Daily-to-half-daily_	1.8	98.2
	σ^2^_Half-daily_	0.0	100.0
	σ^2^_Hourly_	2.1	97.9
	Slope	0.0	100.0
Oxygen	Mean	64.8	35.2
	σ^2^_Total_	0.0	100.0
	σ^2^_Weekly_	0.0	100.0
	σ^2^_Daily_	0.0	100.0
	σ^2^_Daily-to-half-daily_	2.9	97.1
	σ^2^_Half-daily_	2.3	97.7
	σ^2^_Hourly_	0.0	100.0
	Slope	0.0	100.0
pH	Mean	0.0	100.0
	σ^2^_Total_	0.0	100.0
	σ^2^_Weekly_	0.1	99.9
	σ^2^_Daily_	0.0	100.0
	σ^2^_Daily-to-half-daily_	2.7	97.3
	σ^2^_Half-daily_	3.2	96.7
	σ^2^_Hourly_	0.0	100.0
	Slope	0.0	100.0

Results of the variance components analyses (VCA) performed on all the statistics derived from the auto-spectral analyses of the transect stations time-series. Deployment date was fit as a random effect (temporal) and the residual variance is attributed to location (spatial). The VCs were fitted using REML, and many of the VCs approached zero. When fitted using traditional ANOVA estimation, these VCs were negative, indicating a true estimate of zero.

### Spatial Patterns in Time Series Spectra

**Figure 5:**
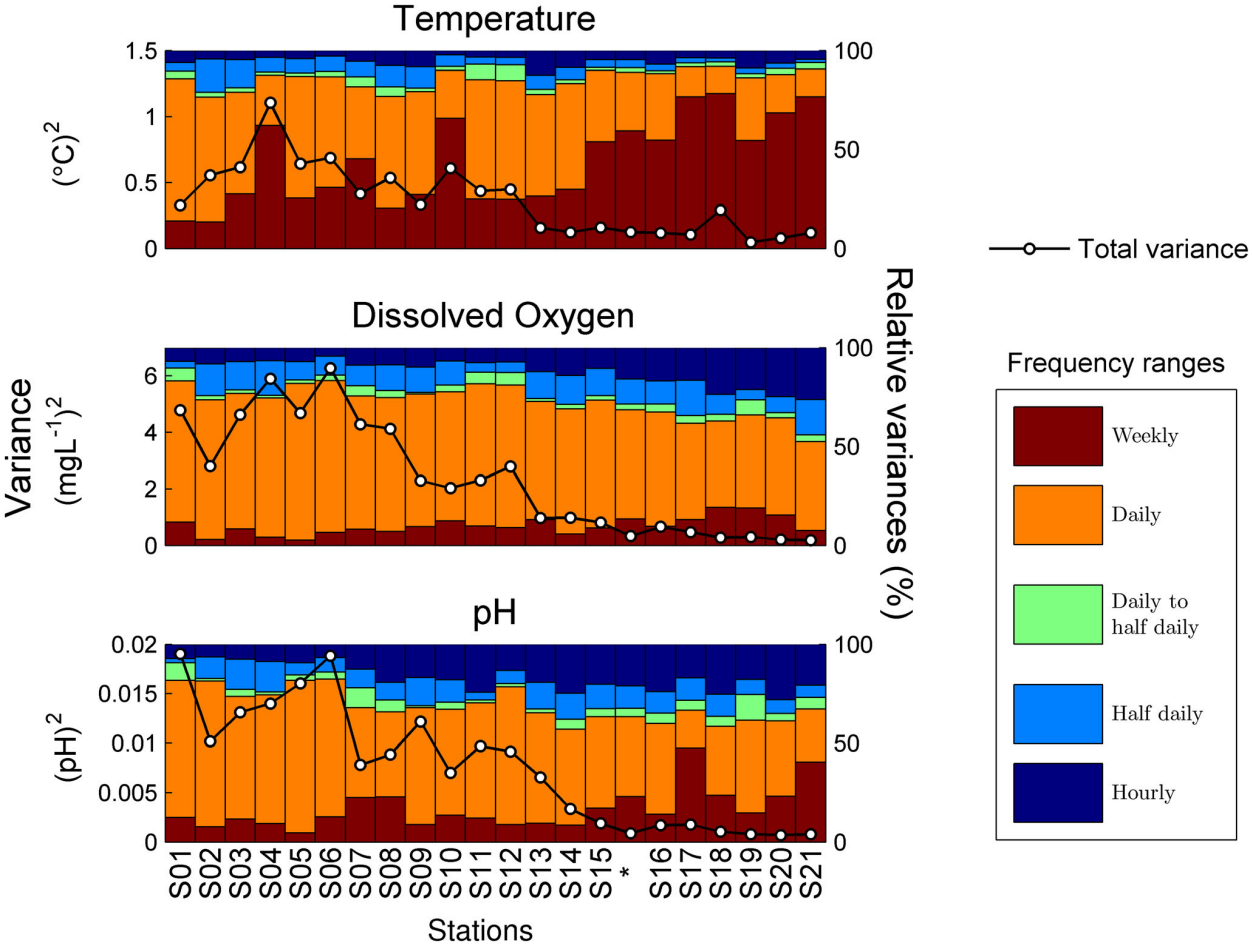
Variance decomposition along the transect. Variance decomposition of temperature, dissolved O_2_ concentration and pH for each one of the deployments along the inshore/offshore transect. Left axes scale absolute variances (black lines), and right axes the relative contribution (in %) of each frequency range to total variance. Sites are ordered from shallowest and closest to shore (S1) to deepest and most offshore (S21). The asterisk marks the average values from the long-term water column station, positioned within the transect according to its depth.

Overall, daily frequencies contained the most variance (Fig. 5), accounting for 42% of total variance in temperature, 63% in [O_2_] and 53% in pH on average. Thus, the relative importance of daily frequencies was higher for biologically-driven parameters (pH and [O_2_]), while the relative importance of weekly frequencies was higher for temperature.

**Figure 6:**
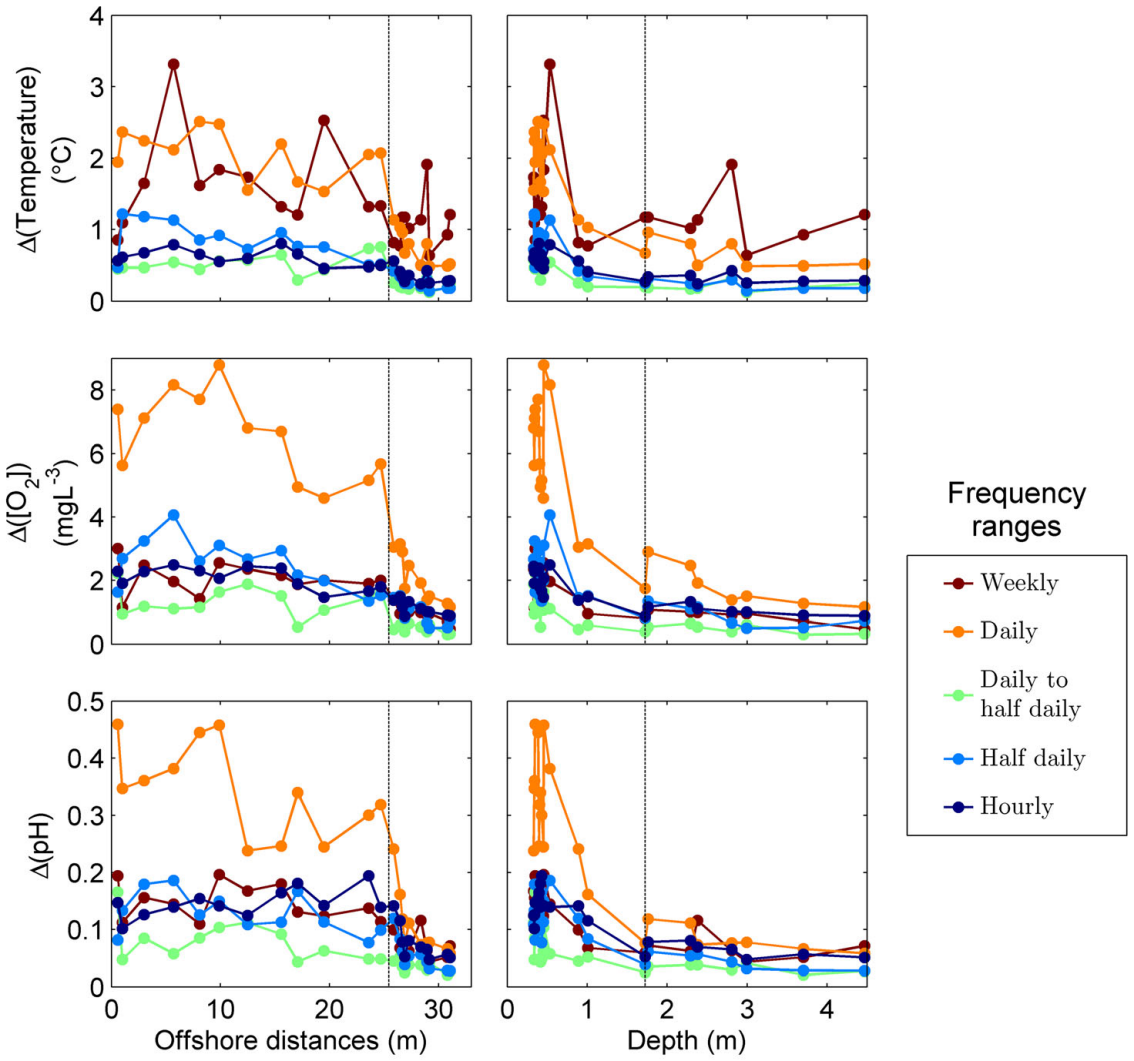
Amplitudes of oscillations by frequency ranges. Amplitudes *A* are calculated from decomposed variances, assuming that all parameters have a normal distribution, as *A* = 4·√σ2. Vertical dash lines mark the position of the long-term time series station.

**Figure 7:**
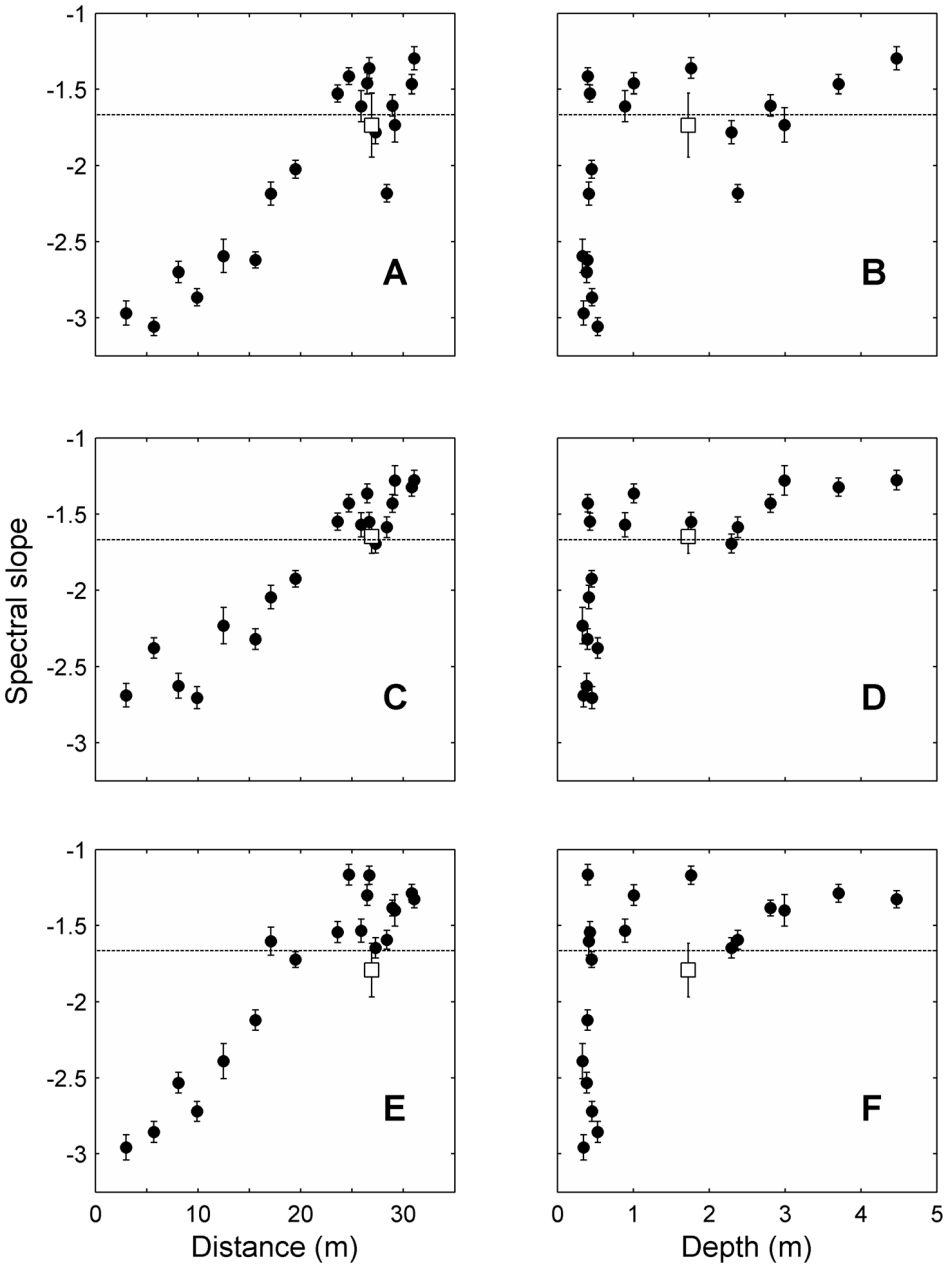
Spectral slopes vs. distance from shore and depth. Spectral slopes of temperature (A & B), dissolved O_2_ (C & D) and pH (E & F) vs. distance from shore (left column) and depth (right column) of each station along the transect. Dots with error bars represent the fitted slopes with 95% confidence intervals. Empty squares with error bars show the mean ± standard deviation of all the spectral slopes in the long-term station estimated for each period of deployment. Dashed horizontal lines mark the −5/3 typical of 3D isotropic turbulence spectra.

Several spatial trends in both total and decomposed variances are observed (Fig. 5). First, total variance of all parameters decreases offshore. Second, the relative contribution of the different frequency ranges changed with the decrease in total variance along the transect. In all 3 parameters, the relative contribution of daily frequencies decreased offshore. By contrast, the relative contribution of weekly frequencies increased with distance to shore, particularly for temperature. Relative contributions of the highest frequencies (>1/10 h^−1^) also increased offshore, but only in the case of the biologically driven parameters (pH and [O_2_]). Finally, distance to shore appeared to be a better linear predictor of changes in all variance components than depth (Figs. 6 and 7).

Spectral analyses provide information about recurrence and persistence of environmental fluctuations (i.e., frequency) vs. their intensity expressed in terms of variance. However, a more natural way to evaluate the contribution of each frequency to overall variability may be achieved by looking at the amplitude of fluctuations. Assuming that values are normally distributed (Fig. 2B), amplitude at a given frequency can be estimated by applying the empirical rule: 95.45% of values lie within 2 standard deviations. Using this approach we estimated, for example, daily oscillations in temperature, [O_2_] and pH to be respectively >2.5°C, >8 mgL^−1^ and >0.45 (compare to Fig. 2A and [14], Fig. 2) in the reef flat, and <0.5°C, ∼1 mgL^−1^, and 0.06 in the reef slope (Fig. 6). Spatial gradients at higher frequencies are less steep, but amplitudes may still be biologically significant.

Finally, spectral slopes also increased linearly with distance to shore (Fig. 7). Close to shore spectral slopes approached −3, whereas offshore the slopes fell between −5/3 and −1. Spectral slopes were negatively related to the daily component of variance, so that the steepest slopes occurred in those sites with highest diurnal variance. This could result from the peaks in daily and half-daily frequencies leaking down to highest frequencies. Note, however, that slopes were estimated after applying a high-pass filter that effectively removed these peaks.

## Discussion

We used two complementary spectral analyses to assess the distribution of temporal variability along a cross-reef transect: variance decomposition at different frequency ranges, and fitting of spectral slopes at the high-frequency end of the spectra. The first assesses the contribution of the most important frequencies to variability; the second is a common way to characterize patchiness in the distribution of a scalar (e.g. [41]) and is better suited to explore the highest frequencies where no individual peaks stand out.

Results show dramatic changes in the regime of temporal fluctuations over spatial scales of meters (Figs. 5, 6 and 7). Even when average values are similar along the transect, as is the case with temperature and [O_2_], individuals on the reef flat experience a highly variable environment, and those on the reef slope a relatively constant one. Here, we first discuss the suitability of the spatial and temporal sampling design and its potential application in other systems; second, we address what physical and biological processes may be driving the observed patterns; and finally, we discuss the ecological relevance of these results in the light of the ongoing discussions about the responses of organisms and ecosystems to natural variability and climate change.

### Sampling Design

Our observational design, combining mobile short-term deployments of sensors and a long-term stationary suite of sensors, made use of limited resources to provide information on the spectral distribution of environmental variance across a spatial transect. Auto-spectral analyses were adequate tools for characterizing variance distribution in this design because variability along the transect was much higher than the long-term temporal dynamics. However, auto-spectra would not be appropriate in systems where differences between sampling periods are pronounced (e.g., at mid and high latitudes where there is strong seasonality). In such cases, an alternative approach would be to normalize each of the two-week time series on the transect to its corresponding period in the long-term station, using cross-spectral techniques. This normalization allows direct comparison of the spectra of fluctuations across transect locations. In our dataset, the spatial patterns obtained using the cross-spectral approach were very similar to the results from the auto-spectral approach we present (see, for example, Fig. 4). This similarity is additional evidence that temporal changes in the regime of fluctuations are small when compared to spatial changes. We chose to present results of auto-spectra instead of cross-spectra, because they are more naturally interpreted, allowing the calculation of the range of values experienced at each location (Fig. 6). However, the use of cross-spectral analyses between a permanent station and a grid of temporary stations may prove valuable in systems where differences between sampling periods are more pronounced than differences across space.

The analyses conducted here assume that the observed spatial patterns in variance are stationary, that is, that ratios between variances at the long-term station and those at each site along the transect remain constant with time. It is a reasonable assumption, given that measurements periods were randomly spread over half a year, and observed spatial patterns remained clear. Again, caution should be exerted when applying this kind of sampling design in systems with strong seasonal and long-term variability or across large spatial scales. In such cases, the use of cross-spectral analyses may prove a better choice.

### Spatial Patterns and their Causes

Spatial and inter-parameter differences occur at all frequencies (Fig. 5). However, a clear pattern is common: all variance components decrease offshore, and spectral slopes get less steep. All statistics are related to both distance to shore and depth (Figs. 6 and 7). Although depth and distance to shore are obviously correlated, the mechanisms by which they may affect the distribution of temporal variability are different.

Depth plays and important role because energy and matter are much more quickly transferred through the shallow water column (<1 m) over the reef flat than through the deeper water column (2–5 m) of the reef slope. This leads to rapid and intense physical, chemical, and biological responses to high-frequency atmospheric forcing on the reef flat. Most physical and biological processes dominant in coastal systems, such as light, tides, waves, or turbulence, are strongly influenced by water column height. Not surprisingly, the relative importance of daily variance decreases offshore whereas that of weekly variance increases (Fig. 5).

Distance to shore reflects horizontal mixing between the reef flat and the bay waters. Limited horizontal mixing may result in long water residence times in the reef flat, depending mostly on tidal oscillations and wind [42]. The fact that the distribution statistics are linearly related to distance to shore, and exponentially related to depth (Figs. 6 and 7) suggests that limited mixing between the reef flat and the reef slope may best explain the observed patterns in variability.

In any case, the combined effects of depth and distance to shore result in clear differences in fluctuation regime between reef flat and reef slope: 1) processes generating variability are most intense and/or amplified in the reef flat, and 2) there is limited mixing between reef flat and slope. The pattern and scale of temporal variability reflect the underlying processes.

At the weekly scale, the longest temporal scale in our sampling, variability is most likely related to weather (e.g. [26]). This is supported by several observations. First, temperature, the most physically-driven and seasonal parameter, shows the largest relative contributions at this scale (Figs. 3 and 5). Second, the time series of weekly variance (Fig. 3) is related to the meteorological record. For example, the highest peak in weekly variance occurs for all parameters in March 2012, during the strongest rainstorm of the study period; this pattern is signaled by a drop in salinity (Fig. 1B). And finally the weekly component does not show clear spatial patterns along the transect, especially for temperature (Fig. 6). For example, a local peak in weekly variance observed in S10 coincides with the rainstorm in March 2012. This lack of clear patterns is to be expected whenever the low-frequency dynamics are confounding the spatial pattern. Kāne‘ohe Bay dynamics are well known to be largely driven by rainstorms [26], [43].

Daily frequencies contributed most to total variance for all parameters (Fig. 5), and decreased offshore, as has been previously described [14]. There are several interdependent physical phenomena with intense diurnal periodicity, including solar radiation, tides, waves and winds. The interplay of these phenomena has been shown to drive daily temperature oscillations both in the water column and inside corals [42], [44] potentially leading to local coral bleaching conditions. On the other hand, daily cycles of net photosynthesis/respiration, combined with the depth and limited horizontal mixing outlined above, are likely to drive the variance of daily frequencies in [O_2_] and pH (e.g. [12]). Although the biomass of corals and algae is higher on the reef slope than the reef flat, the limited mixing on the reef flat may amplify the effects of the photosynthesis/respiration cycle observed in the water column, contributing to the stronger diurnal cycle on the reef flat.

Finally, at the highest frequencies of our sampling, turbulence is likely to be the most important factor contributing to variability because it is the main mechanism transferring variance from large to small scales and will cause mixing of scalars between reef flat and slope. A scalar under isotropic turbulence is expected to show a spectral slope of −5/3 (e.g. [5], [41], [45]–[46]). Thus, the average −5/3 spectral slope at the long-term station (Fig. 5) suggests that turbulence may be important at these scales. However, it is unclear whether the sampling rate (0.1 min^−1^) was fast enough to resolve any part of the turbulent inertial subrange [41]. It was certainly too slow to resolve the smallest scales, usually ranging between milliseconds and minutes [47]. But it could still be faster than the overturning rates of the largest eddies, and thus spectral analyses could overlap with the inertial subrange. To elucidate this, we assumed that the size of the largest turbulent eddies was limited by the height of the water column, and applied Taylor’s frozen hypothesis on the horizontal velocities from the ADCP [48]. This heuristic approach yields scales between a few minutes to about 5 hours in the reef slope, and between tens of seconds to less than 5 minutes in the reef flat. Thus, some overlap with the inertial subrange may occur in the reef slope, but not in the flat.

Spectral slopes offshore lie between −5/3 and −1. These values are indicative of turbulent diffusivity driving changes at these scales. However, slopes gradually decreased onshore, to reach values of −3 at the sites nearest to shore. This indicates that physical or biological processes other than water motion are driving variability [5]. Spectral slopes are not by themselves good diagnostics of the mechanics of a system [41], especially in complex, highly energetic environments like the reef flat, where many phenomena, including breaking waves, tides and currents, are interacting. Nonetheless, they provide an easy way to parameterize environmental variability as experienced by organisms. Water motion at these scales, whether turbulent or not, is critical for organisms because it controls the transport and exchange of nutrients and particles not only horizontally but also between water column and bottom [49]. The acute patterns in the spectral slopes between reef flat and reef slope are thus symptomatic of fundamental differences in the environment organisms are sensing, and deserve to be studied in connection to physiological responses.

### Ecological Relevance

We have shown robust spatial patterns in the regime of environmental fluctuations across a reef flat at relatively high frequencies. But, to what extent do changes occurring at these frequencies impact the life of reef organisms, and what role do they have in defining their spatial distribution? Such questions are obviously out of the scope of the present study, which solely deals with environmental variability. However, our approach may be useful in defining the limits of natural variability that individuals are experiencing, thus allowing for hypothesis-driven field studies, and providing a basis for better predictions of biological responses to change.

The amplitudes of oscillations in temperature, pH and oxygen observed at short timescales on this small transect are comparable to long-term trends (Table 2). At the long-term station (Fig. 1B), seasonal oscillations in temperature (∼6°C) and pH (∼0.19) are comparable to daily oscillations on the reef flat. The increase in temperature (0.74°C±0.18°C) and the decrease in pH (0.1) attributed to climate change over the past centuries [50]–[51], are comparable to changes we observe at hourly frequencies, for example. These hourly variations of pH and temperature are within the range known to impact coral bleaching and growth [52]–[53]. Of course, perturbations that are too short and/or infrequent may not trigger a response in organisms [54] or have a lasting effect. But natural variability provides the context for ongoing discussion about acclimatization and response of organisms and ecosystems to climate change [15].

**Table 2:** Amplitude of fluctuations at different frequency ranges.

	Climate change	Seasonal signal	Weekly	Daily	Daily tohalf-daily	Half-daily	Hourly
ΔTemperature (°C)	0.74±0.18	∼6	0.6–3.3	0.5–2.5	0.1–0.7	0.1–1.2	0.2–0.8
Δ[O_2_] (mgL^−1^)	–	∼1.5	0.5–3.0	1.2–8.8	0.3–2.2	0.5–4.1	0.9–2.5
ΔpH (units)	0.1	∼0.19	0.04–0.20	0.06–0.46	0.02–0.17	0.03–0.19	0.05–0.19

Amplitude of oscillations at particular frequency ranges derived from the partition of variances performed with spectral analyses, assuming normal distributions and applying the empirical rule. The amplitude of climate change for temperature corresponds to the estimated changes between 1906 and 2005 [51]. The climate change value for pH corresponds to the decrease in surface pH between 1750 and 1994 derived from estimated uptake of anthropogenic CO_2_ by the ocean [52]. Seasonal signals are derived from the long-term time series.

For example, corals can acclimatize to changes in fluctuation frequency [21], [55]–[56], and exposure to high-frequency low-intensity fluctuations may increase tolerance, through acclimatization, to large environmental events [21]. On the other hand, acclimatization of organisms to long-term changes can be diminished by exposure to high-frequency intense fluctuations [57]. Further, our own data shows that these spatial gradients in pH variance are correlated with bioerosion/accretion patterns (Silbiger et al., unpublished data).

Spatial patterns in high-frequency environmental variability could also translate into different distributions of biological organisms and processes. Temporal and spatial variances are correlated across scales (e.g. [58]). Frequent, short, and weak perturbations tend to be local, whereas intense perturbations usually impact large areas for long periods. Thus, temporal variance at high frequencies can potentially be very different in two sites that are spatially very close. Immediate responses to high-frequency changes in the environment may be small, hardly detectable shifts in organism function. However, in the long run, these fluctuations may affect species distributions and shape biological spatial heterogeneity and resilience to large environmental change. Thus, it is likely that the differences in exposure to environmental fluctuation across the reef are driving patterns of genetic and community structure. New molecular tools that allow measuring individual responses as changes in gene and protein expression or enzyme activity (e.g. [8], [55]) offer a possibility to explore the question of how these responses can translate into long-term spatial changes in community structure.

These are important issues to address as global climate change and ocean acidification alter the pattern of environmental fluctuations. Experimental works have mostly addressed how changes in baselines may affect individuals and populations, but there is an increased awareness of the importance of variance at organism scales (e.g. [13], [21], [56], [59]–[63]). Under climate change scenarios we may expect dispersion statistics (variances, ranges or extremes) to be altered by: 1) an increase in the frequency of intense meteorological events [11], and 2) a multiplicative effect on variances (i.e. variance correlates with mean, as opposed to independent variance typical of additive models). Multiplicative changes in mean temperature and pH are more likely to lead to ranges of variability outside tolerance limits for many of these coral reef organisms. Biologically reactive parameters with nonlinear responses to environmental changes can be hypothesized to show multiplicative variances [64].

## Conclusions

The sampling scheme that we used allowed us to explore the spatial distribution of temporal variability making use of limited resources. Spectral analyses of time series reveal patterns in the regime of environmental fluctuations at all frequencies resolved in the study that occur over very short distances making it a good model to explore the acclimatization and adaptation of organisms to high-frequency variability.

Furthermore, the spectral analyses allow a quantification of the amplitude of fluctuations occurring at specific scales. The results show that the fluctuations at relatively high frequencies and also at weekly scales are comparable in magnitude to diurnal, seasonal and longer trends (Table 2). This pattern is likely particular to low latitudes with mild seasonality, but it stresses the importance of looking at the whole spectrum of natural variability rather than focusing on specific frequencies.

In general, spectral analyses cannot by themselves distinguish the underlying mechanisms that generate environmental variance. They can, however, reveal and quantify patterns in variance that organisms in different places will experience. Whether and how organisms may sense and respond to these different regimes of fluctuations, and whether this can translate into long-term spatial changes in community structure, can now be addressed *in situ* with new molecular tools (e.g. [9], [18], [56]). The analyses presented here show that coastal systems, and coral reefs in particular, may be good systems to investigate *in situ* responses to high frequency fluctuations, and provide clues about what scales are worth exploring.
